# Forwarding Techniques for IP Fragmented Packets in a Real 6LoWPAN Network

**DOI:** 10.3390/s110100992

**Published:** 2011-01-18

**Authors:** Alessandro Ludovici, Anna Calveras, Jordi Casademont

**Affiliations:** Wireless Network Group (WNG), Universitat Politècnica de Catalunya, C/Jordi Girona 1-3, Mòdul C3, 08034 Barcelona, Spain; E-Mails: anna.calveras@entel.upc.edu (A.C.); jordi.casademont@entel.upc.edu (J.C.)

**Keywords:** Wireless Sensor Networks, 6LoWPAN, performance evaluation, mesh under, route over

## Abstract

Wireless Sensor Networks (WSNs) are attracting more and more interest since they offer a low-cost solution to the problem of providing a means to deploy large sensor networks in a number of application domains. We believe that a crucial aspect to facilitate WSN diffusion is to make them interoperable with external IP networks. This can be achieved by using the 6LoWPAN protocol stack. 6LoWPAN enables the transmission of IPv6 packets over WSNs based on the IEEE 802.15.4 standard. IPv6 packet size is considerably larger than that of IEEE 802.15.4 data frame. To overcome this problem, 6LoWPAN introduces an adaptation layer between the network and data link layers, allowing IPv6 packets to be adapted to the lower layer constraints. This adaptation layer provides fragmentation and header compression of IP packets. Furthermore, it also can be involved in routing decisions. Depending on which layer is responsible for routing decisions, 6LoWPAN divides routing in two categories: mesh under if the layer concerned is the adaptation layer and route over if it is the network layer. In this paper we analyze different routing solutions (route over, mesh under and enhanced route over) focusing on how they forward fragments. We evaluate their performance in terms of latency and energy consumption when transmitting IP fragmented packets. All the tests have been performed in a real 6LoWPAN implementation. After consideration of the main problems in forwarding of mesh frames in WSN, we propose and analyze a new alternative scheme based on mesh under, which we call controlled mesh under.

## 1. Introduction

Progress in both wireless communication technologies and in miniaturization of electronic devices has led to a rapid growth and diffusion of Wireless Sensor Networks (WSNs). The nature of wireless communications enables WSNs to be developed in all kind of environments. WSNs have virtually no limitations in the fields where they can be applied, and have found use in many different areas, such as industrial control and monitoring, traffic and vehicular control, habitat and environmental monitoring or health monitoring. In all their multiple applications, WSNs are able to interact with the surrounding environment not only collecting information, but also reacting to certain events.

WSNs are usually composed of hundreds of low-power and low-cost devices. These are characterized by having constrained resources and very limited capabilities as well as short communication ranges. The operational constraints of a WSN and its nodes are the critical aspects that influence the choice of one protocol stack or another. Regarding the physical and data link layers, a widely used solution is offered by the IEEE 802.15.4 standard [1]. WSNs using this standard are defined as Low Power Wireless Personal Area Networks (LoWPANs). Network nodes in LoWPANs are characterized by having low data rates of 250 Kb/s in the 2.45 GHz frequency band, a 8-bit or 16-bit CPU, 4 KB to 8 KB of RAM and 48 KB to 128 KB of ROM. Nodes are usually battery powered and the communication range is reduced to a maximum of 10 meters when transmitting with the maximum output power. Crossbow’s TelosB mote [2] is a typical example of a low-cost wireless sensor used in LoWPAN. It features 16-bit RISC MCU at 8 MHz and 16 registers. The platform offers 10 kB of RAM, 48kB of flash memory and 16 kB of EEPROM. TelosB motes have been used as a hardware platform to develop our test-bed network.

While the IEEE 802.15.4 is nowadays a standard for the lower protocol layers of WSNs, problems arise when approaching upper layers. In fact, the growing interest around WSNs has led to the creation of different communication protocol proposals. This variety of solutions has limited the possibility to interconnect and integrate various WSNs based on different network protocols. The adoption of the IPv6 protocol as the network layer has been proposed to overcome these problems. The original proposal was made by a specific IETF working group created with the aim of implementing the IPv6 protocol over LoWPAN [3]. The resulting protocol stack goes under the name of 6LoWPAN [4].

Specifications on how to support transmission of IPv6 packets over LoWPAN and meet the IPv6 requirements have been defined in the RFC 4944 [5]. An intermediate layer between network and data link layers, known as the adaptation layer, has been created to enable IPv6 datagrams to be conforming to the lower layer requirements. Actually, in IPv6 specification [6] the MTU is fixed to 1,280 bytes, while the MTU defined for IEEE 802.15.4 to 127 bytes [1]. The adaptation layer provides fragmentation and reassembling of IPv6 packets as well as header compression. Fragmentation of the IPv6 datagram is necessary to meet the MTU specification of the 802.15.4 standard, while the header compression is required to reduce the space consumed by the 40-byte length IPv6 header. Finally, the adaptation layer can also be involved in forwarding decisions. Depending on which layer is in charge of routing decisions, 6LoWPAN classifies routing into two categories: In mesh under the layer of interest is the adaptation layer, while in route over it is the network layer.

In this paper, we analyse both routing schemes focusing on how they forward 6LoWPAN frames. We consider 6LoWPAN communications requiring IP fragmented packets. The analysis is conducted through a performance evaluation of mesh under and route over in terms of latency and energy consumption. For our purpose, we develop and test both solutions in a real 6LoWPAN implementation. Moreover, in this paper we present a new routing proposal based on mesh under. Our proposal seeks to improve the mesh under fragment processing by adding control on the fragment forwarding process. We also consider an alternative route over scheme already implemented in the 6LoWPAN software solution we adopt in this paper.

The paper is organized as follows. First, in the following section we present the related work. Then, in Section 3 we discuss the existing 6LoWPAN routing techniques and present our proposal. In Section 4 results and discussion of the performance evaluation are reported. Finally, in Section 5 we conclude the paper.

## 2. Related Work

Previous work by the authors [7] examined mesh under and route over in 6LoWPAN communications not requiring IP packet fragmentation. We tested both solutions in a multi-hop scenario, evaluating their performance in terms of end-to-end delay and round-trip time. From the tests we conducted, mesh under turned out to have better latency performance than that achieved by route over. Forwarding packets at adaptation layer avoids the hop-by-hop compression/decompression of the IPv6 header, resulting in less time spent by mesh under to forward the packet.

An analytical evaluation of mesh under and route over was presented in [8]. Both solutions have been compared using a probabilistic model. As a 6LoWPAN scenario, a multi-hop network is assumed where communications require IP packet fragmentation. Results in [8] demonstrated that route over has a higher fragment arrival probability than mesh under. Furthermore, it has been shown that route over can experience buffer overflow when the traffic generated in the network is high and a node receives packets from different paths. Analysis on latency demonstrated that it is higher in route over.

An evaluation of different 6LoWPAN implementations was carried out in [9]. From all the implementations analyzed, the only one supporting both mesh under and route over techniques is Blip [10]. Blip is the 6LoWPAN implementation we adopt for this work. Details on Blip are given in Section 4. Silva *et al*. tested the considered implementations, evaluating how they perform considering RAM and ROM usage, time required to send a packet and energy efficiency. All the tests have been done considering different packet sizes. Blip demonstrated that it scaled well in energy efficiency and ROM usage. Actually, Blip gave the poorest performances considering RAM usage. In fact, while the other implementations have a constant use of RAM, Blip requires an increasing amount of memory when incrementing the packet size.

Finally, route over and mesh under solutions for 6LoWPAN are discussed in [4] and [11]. In particular, in [11] a series of guidelines for 6LoWPAN routing are specified, including both mesh under and route over solutions. In [4], an extended explanation of the adaptation layer and issues of mesh under and route over are presented.

## 3. Forwarding Strategies in 6LoWPAN

As mentioned, 6LoWPAN divides routing techniques into mesh under and route over. The distinction is based on which layer of the 6LoWPAN protocol stack is in charge of routing decisions; in route over they are taken at the network layer, and in mesh under at the adaptation layer. The main difference between these two schemes depends on how the packets or fragments are processed before being forwarded. Besides these basic techniques, we also consider two alternative routing techniques to improve performance of fragment forwarding.

Regarding route over, an alternative scheme is explained in [12]. We refer to it as enhanced route over. This proposal seeks to avoid the hop-by-hop fragments reassembling by establishing a virtual circuit between the source and the destination nodes of the fragmented packet.

**Figure 1.**
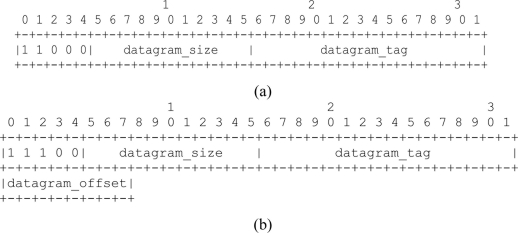
6LoWPAN Fragment headers. **(a)** First fragment; **(b)** Subsequent fragment.

Concerning mesh under, we observed that it was particularly affected by a high number of retransmissions and a consequent growth of the packet loss percentage. We found the main cause in the absence of control on the fragment forwarding process. Actually, mesh under is not able to distinguish if the frames to be forwarded are part of an IP fragmented packet or not. Consequently, if a fragment is dropped, then the subsequent fragments are forwarded, although it is not possible to reconstruct the packet. This results in a waste of bandwidth. To overcome this problem, we propose a new approach to mesh under that enables the forwarding process to be controlled by monitoring the fragmentation header (Figure 1). We refer to this approach as controlled mesh under.

In this work, we centre our attention on these different routing techniques and give an evaluation of the consequences they have on WSN performance. It should be pointed out that enhanced route over is the only routing technique implemented in Blip. In this work, we implement the remaining techniques in Blip. To the best of our knowledge, this is the first evaluation of 6LoWPAN routing techniques dealing with IP fragmented packets in a real network implementation. Below, we describe the studied forwarding techniques.

### 3.1. Route Over

Since the 6LoWPAN frames are forwarded at the network layer, it is necessary that the adaptation layer processes the received frames in order to recreate the original IP packet. This operation occurs at each hop [12].

Should the received frames not be part of a fragmented IPv6 packet, they will only need to be passed to the network layer and then processed by the routine responsible for unpacking the compressed IPv6 header. Should the packet need to be forwarded, the routine responsible for route over forwarding will look at the routing table in order to choose what the next-hop will be. The packet then goes back to the adaptation layer, which will compress the IP header again and send it.

As previously anticipated, if the received frames are part of the same IP fragmented packet, the adaptation layer first needs to reassemble them in order to reconstruct the original packet. Hence, all the incoming fragments are stored in a proper buffer and the reconstruction process starts only when the last fragment arrives. Once reconstructed, the original IP packet will be passed to the network layer. If the packet has to be forwarded, the forwarding routine will process and send it back to the adaptation layer. Finally, the IP packet will be fragmented again and its fragments will be sent to the next-hop. These operations are performed in each node the packet goes through before reaching its destination.

The maximum IP packet size allowed in 6LoWPAN corresponds to 1,280 bytes, which is the IPv6 MTU. When approaching the maximum allowed size, there is the possibility of buffer overflow in the nodes that are receiving packets to forward. Even if buffer overflow does not occur, the high work load required to store and reassemble the packet could significantly slow down the forwarding of fragments through the network. This would increase latency and the energy consumed by the device.

When the adaptation layer fragments the IP packets, it appends in each 6LoWPAN frame a header indicating whether the frame is the first fragment or one of the followings. Figure 1(a) shows the fragmentation header for the first fragment, while in Figure 1(b) the fragmentation header for the subsequent fragments as specified in [5].

With reference to Figures 1(a,b), the first four bit of both headers indicate the dispatch values that the adaptation header will check to identify what kind of fragment it is dealing with. Datagram_size field uses 11 bit to encode the size of the entire IP packet before fragmentation. The value of this field must be the same for all the fragments composing the IP packet. The 16-bit length datagram_tag field identifies that a sequence of fragments is part of the same IP packet. The 8-bit field datagram_offset is defined only for subsequent fragments. It specifies the offset, in module of eight bits, of the fragment from the beginning of the payload datagram.

### 3.2. Enhanced Route Over

The major drawback of route over is the hop-by-hop fragment reassembly. This characteristic can significantly increase latency and increase the energy required by a node to forward a packet. However, different solutions can be applied to route over in order to solve this problem. In fact, route over could be implemented with methods able to create virtual reassembly buffers that remember just the IPv6 header contained in the first fragment [13]. In [12] the creation of a state associated to the IPv6 source address and to the datagram tag (Figure 1) is proposed. This information is contained respectively in the IPv6 header and in the fragmentation header carried by the first fragment. This solution allows a virtual circuit to be established for the subsequent fragments. As mentioned earlier, Blip adopts a route over alternative solution based on this proposal.

In nodes implementing enhanced route over, the adaptation layer checks the fragmentation header of each incoming frame. Should the fragment be recognized as the first, it is sent to the IP layer to unpack the IPv6 header. Should the fragment need to be forwarded, the node gathers the information required to create the state associated to forwarding and establishes the virtual circuit. This information is contained in the IPv6 header and in the first fragment header. When each subsequent fragment reaches the node, this is forwarded through the virtual circuit without the need to check any routing table. The virtual circuit is deactivated and the state associated erased from memory after the last fragment has been forwarded.

### 3.3. Mesh Under

**Figure 2.**
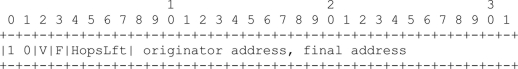
6LoWPAN Mesh header.

In mesh under, packet forwarding is transparent to fragmentation. The adaptation layer treats each incoming packet or fragment in the same way. There is no control of the 6LoWPAN fragmentation headers. To forward a packet or a fragment, the adaptation layer combines the information contained in the mesh header with the source and destination addressees carried in the IEEE 802.15.4 header. In this way, the IPv6 header does not need to be unpacked. When sending packets or fragments, the adaptation layer adds a mesh header (Figure 2) to the 6LoWPAN frame indicating that it should be handled with mesh under. It is mandatory that the mesh header be appended at the beginning of the header chain [5].

Should the received frame be recognized as mesh frame, the mesh under routine will obtain the information contained in the mesh header; that is, the originator and final address and the hop limit. Should the received frame need to be forwarded, the mesh header information and the destination address contained in the IEEE 802.15.4 header are passed to the mesh under forwarding routine, which will return the IEEE 802.15.4 address of the next hop to the mesh under routine. Once the mesh under updates the hop limit field, the frame is ready to be forwarded to the next hop. All the forwarding process is performed without ever leaving the adaptation layer.

Figure 2 shows the mesh header format as specified in [5]. The mesh header dispatch value is specified by the first two bits, set to 1 and 0, respectively; V and F bits indicate the length of the originator and final addresses. If they have the value of 0, the addresses are IEEE extended 64-bit addresses; if the value is 1, they are short 16-bit addresses. Originator and final addresses are the address of the node starting the communication and its destination, respectively. The remaining four bits of the first octet indicates the number of hops. It can be defined up to 14 hops. An extra octet can be added to define a number of hops greater than 14 by setting all the 4-bit of hop left to 1.

### 3.4. Controlled Mesh Under

Since in the mesh under forwarding process there is no control on the frames to be forwarded, unnecessary fragments may be propagated. In fact, if any fragment gets lost before it reaches the destination then the rest of fragments will be forwarded unnecessarily. In this case, the whole packet cannot be reassembled. Even though no fragment is lost, they may still arrive at destination out-of-order. This would complicate the reconstruction process at the destination node. Adding control on the mesh under forwarding process would reduce the probability of out-of-order delivery of fragments, and the transmission of useless fragments would thereby be avoided. This would result in a better use of the bandwidth and in a simplification of the fragment reassembling, thus allowing low complex and less resource demanding code to be developed.

Our controlled mesh under proposal seeks to solve these problems. We propose adding a control to the mesh under forwarding process that allows the fragmentation header of the incoming fragments to be monitored.

The control starts each time a node receives a mesh frame containing the first fragment header. It begins by storing the information contained in the first fragment and mesh headers. In more detail, this information is relative to the tag and the size fields of the first fragment header and the originator address of the mesh header. This information allows us to determine if the subsequent packets are part of the same IP fragmented packet. Should the subsequent fragments belong to the same packet, then the controlled mesh under verifies if the reception is in-order. This is established by checking the offset field of the fragmentation header. Should the fragment be the one expected, the forwarding routine will start the process to forward the fragment. This corresponds to the mesh under forwarding process. If the received fragment does not match the one expected, then the previous node is asked to retransmit the expected fragment. When the correct fragment has been received, the forwarding process can be resumed. Should the fragment not be received, the forwarding process is cleared and subsequent fragments will not be forwarded, thereby avoiding bandwidth waste.

## 4. Results and Discussion

In this section, we report the performance evaluation done for the four routing schemes presented in this paper. We compare the different forwarding strategies of mesh under, route over, controlled mesh under and enhanced route over focusing on the obtained performance in terms of latency and energy efficiency. For latency, we first consider the average round-trip time delay time obtained by pinging a node with different payload sizes. We then evaluate the average end-to-end delay time obtained by transmitting UDP packets according to different payload sizes and network topologies.

For round-trip time tests we considered a two-hop network formed by a base station sending ping requests to a node located at a distance of two-hop. We considered this distance sufficient to give a correct round-trip time performance evaluation and to appreciate the different effects each forwarding strategy has on it.

**Figure 3.**
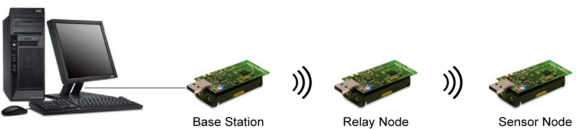
Topology for a two-hop network. In round-trip delay time tests, the base station sends ping requests to the sensor node. In end-to-end delay time tests, the sensor node originates the UDP packet flows. Current consumption is measured in the relay node.

The base station acts as a border router and bridge between the serial and radio link. It was plugged via a USB port to a computer running a Linux operating system. Forwarding of ping requests and responses were executed in the relay node. Figure 3 shows the network topology for a two-hop scenario. The output power of the sensors was fixed to −25 dBm, while distances between sensors were fixed to 20 cm. The chosen values for power and distance proved to be sufficient to create a multi-hop network.

In the end-to-end delay tests, the base station was the destination of the fragments, while the source was located in the sensor node. In this work we consider various scenarios in which this node is at a distance ranging from two to four hops. The topology of the network used as test-bed reflects possible applications of a WSN requiring a small number of nodes. Possible applications can be found in the healthcare domain, where sensors are in charge to monitor the medical parameter of a patient and report the results to a base station. Another example can be found in the sports domain, where the WSN could be used to monitor the performance or the training of an athlete. Nevertheless, this limited topology is sufficient to test the forwarding strategies of the routing techniques discussed in the paper.

The end-to-end delay time tests seek to emulate a possible application scenario where a sensor is in charge of monitoring a certain environment variable and periodically reports the collected values. End-to-delay results do not take into account the time the base station requires to process the incoming packets. In the average round-trip time evaluation, results are strongly influenced by the time the base station spends elaborating and passing the ping response to the operating system. We estimate this time to be in the order of 178 ms. Further delay is introduced by the operating system to generate the ping request and pass it to the base station.

As a software solution, we use an open-source TinyOS based 6LoWPAN implementation developed by the University of California at Berkeley called Blip [10]. Blip implements a multi-path routing algorithm. Consequently, each node maintains multiple next-hop entries for any given path. Different fragments of the same IP packet may take different paths through the network. In this way, the routing algorithm may influence results. Since our aim is to evaluate the forwarding strategies only, we should prevent the collected results from being altered from the routing algorithm performances. We solve this problem by using static routes in which each node has two default next-hop entries selected, depending on the destination address of the mesh header or the IPv6 header. Moreover, it should be pointed out that only mesh under and route over would work with multi-path routing algorithms. Both controlled mesh under and enhanced route over must use the same path to forward all the fragments. In fact, if a fragment could be forwarded to multiples next hop, then we could not create either any state associated with forwarding or ensure the in-order delivery of fragments. However, creating a state associated in the source node would allow the use of these alternative solutions, although it would not be possible to use any multi-path forwarding.

As mentioned earlier, Blip implements the enhanced route over routing scheme. Although Blip supports mesh under, only the functions to interact with the mesh header are implemented. We develop the appropriate code and modify some of the existing codes to enable mesh under, controlled mesh under and route over in Blip.

### 4.1. Round-Trip Delay Time Evaluation

**Figure 4.**
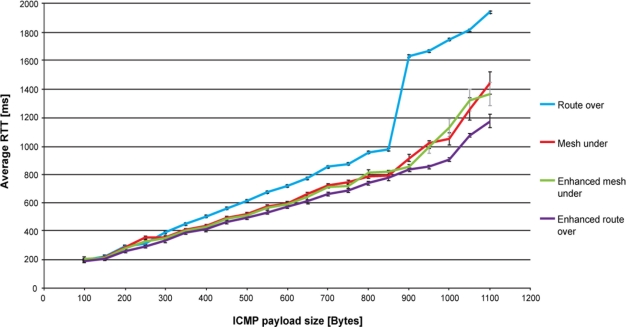
Round-trip delay time evolution according to ICMP payload size. Buffer congestion affects route over when reaching a payload size of 900 bytes, causing the big jump in the average round-trip delay time.

Figure 4 shows the round-trip time performance for route over, mesh under, controlled mesh under and enhanced route over. Each point in the graph represents the average value of 100 ping responses that successfully reached the destination. Payload size ranges from 100 to 1,100 bytes with increments of 50 bytes. The number of fragments goes from 2 for a 100 bytes payload, up to 12 for 1,100 bytes. The reported average values have a confidence level of 95%. Each ping request is sent as soon as the preceding ping reply is received.

As expected, route over has the worst performance. Reassembling and fragmenting packets at each hop slows down the communication, especially when the payload size is high and more fragments are involved in the communication. However, this feature of route over enables the standard deviation to be reduced, resulting in the routing scheme having the lowest deviation. Because of the high number of retransmissions affecting mesh under, and to a lesser extent controlled mesh under and enhanced route over, the standard deviation for both mesh techniques is quite high, while it remains quite low for enhanced route over.

**Figure 5.**
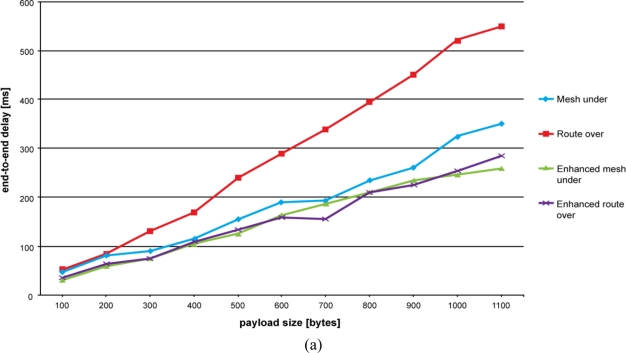

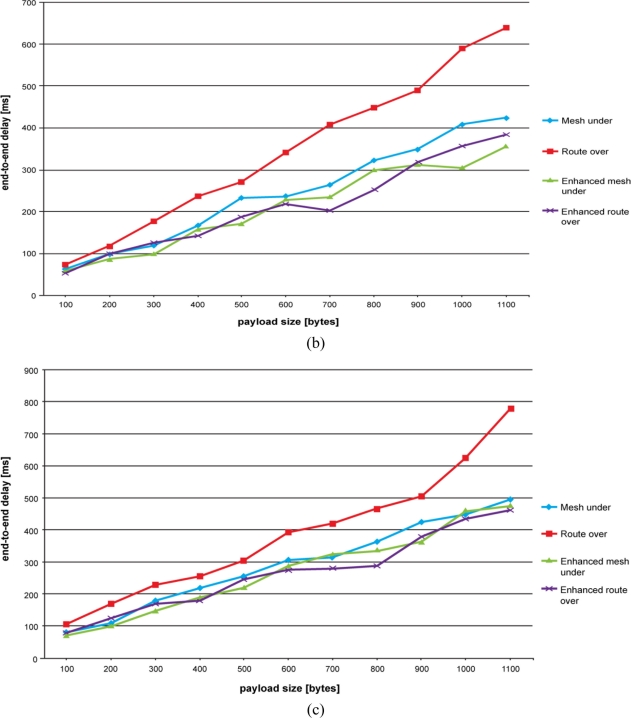
End-to-end delay time evolution. The number of retransmissions is lower in controlled mesh under than in mesh under, resulting in a better end-to-end delay time trend. **(a)** End-to-end delay time for a two hops network. **(b)** End-to-end delay time for a three hops network. **(c)** End-to-end delay time for a four hops network.

In Figure 4 one may observe that the trend of round-trip time has almost a linear evolution for each considered solution. However, when approaching the maximum packet size this trend changes quickly. In particular, the round-trip time performance of route over rapidly gets worse, between 800 and 900 bytes. This is explained by the fact that the buffer capacity is reaching its maximum, which causes memory congestion. Moreover, by increasing the RAM usage as the packet size augments [9], Blip leaves a very limited space to perform the packet processing required by route over. We found this behaviour to be a major cause of memory congestion. Furthermore, we found that memory congestion occurs when the relay node was subjected to an uninterrupted packet flows, such as the one generated by ping requests and response. In fact, spacing the packet transmission, as is done for end-to-end delay evaluation (Figure 5), solved this problem. Regarding mesh under and controlled mesh under, the worsening of round-trip time is explained by observing that the number of retransmitted fragments becomes high in comparison with the other techniques. Enhanced route over has the best round-trip time performances. However, for payload size lower than 900 bytes, the performance is very similar with that obtained in controlled mesh under, while improving it for higher payload size.

The buffer problems affecting route over also influence the packet loss percentage results. As shown in Table 1, from a payload size of 900 bytes to 1,100 bytes, the packet loss percentage obtained in route over becomes of the order of magnitude of the other routing techniques. For lower payload sizes route over proves to be more robust to packet loss, as expected. The performance in packet loss of enhanced route over compared with that obtained by route over shows a worsening of the packet loss. Respect to enhanced route over, controlled mesh under has a better packet loss up to 500 bytes, while for higher payload size this loss becomes similar.

The control on fragment forwarding provided by controlled mesh under improves the packet loss performance with respect to mesh under, which has the worst packet loss percentage. In fact, controlled mesh under avoiding the propagation of unnecessary fragments enables channel occupancy to be lowered. In this way, it is subjected to less retransmissions caused by collisions and consequently to a lower packet loss. The major cause of packet loss in mesh under, controlled mesh under and enhanced route over is found in retransmissions caused by collisions. Collisions occur because the relay node is continuously subjected to fragment reception and has to forward them instantly. In this scenario, the relay node may not detect the reception of a fragment if it is occupied in forwarding another one. As a consequence, the node that was transmitting the dropped fragment will retransmit it to the relay node. It should be pointed out that the retransmission policy used in Blip drops fragments after a maximum of five retransmissions. On the other hand, collisions do not affect route over, since a node using this technique has to wait until the reception of the last fragment to start reconstructing the packet and begin the forwarding process.

Returning to the round-trip delay time performance, a main cause of the worst performances of route over is found in the time elapsed between the reception and forwarding of a fragment. Actually, a node implementing route over is forced to wait until the reception of the last fragment before forwarding the first. We estimate that for a payload size of 1,000 bytes, the time elapsed between the reception and the forwarding of the first fragments is in the order of 125 ms. This value corresponds to the time spent by the previous node to set up and send all the fragments composing the original packet. In mesh schemes and in enhanced route over, the forwarding is immediate to the reception of the fragment. It only takes the time to process the fragment. In previous work [7], we estimated this time to be 11 ms with a standard deviation of 2.1 ms. A further delay is due to the compression/decompression of the IPv6 compressed header. However, the order of magnitude of this delay [7] is not comparable with that introduced by packet reconstruction or fragmentation.

Mesh under and controlled mesh under performance under looks very similar. It seems that the control process that controlled mesh under executes for fragment forwarding does not lessen its performance, or produce any significant enhancement with respect to mesh under. On the other hand, enhanced route over shows considerable improvement with respect to route over. As expected, latency decreases significantly, avoiding hop-by-hop fragments reassembling.

### 4.2. End-to-End Delay Time Evaluation

Results of end-to-end delay time evaluation are shown in Figures 5(a–c). As explained above, our aim is to emulate an application scenario where a node is sensing a certain environmental variable and periodically reports its value to the base station. This period has been fixed to 5 seconds.

As can be seen in Figure 5, route over confirms its negative trend. Controlled mesh under, mesh under and enhanced route over have the lowest end-to-end delay time, with the former having the best performance. Once again, the high number of retransmissions that occurs in mesh under makes the difference when compared with controlled mesh under. The alternative route over scheme significantly improves the end-to-end delay performance by avoiding hop-by-hop fragment reassembling.

On augmenting the number of hops, we observe that the end-to-delay performance for mesh under and controlled mesh under become similar. This can be especially appreciated for higher payload sizes. Contrary to our expectations, increasing the number of hops does not augment the difference between route over and mesh under. In fact, the more hops we have the more retransmissions are required to propagate fragments with a mesh under technique. This makes the route over trend approach mesh under, controlled mesh under and enhanced route over trends. However, differences become more significant for higher payload sizes. As experienced previously, route over gets worse when the number of fragments composing the packet becomes high and the buffer is approaching its limit.

### 4.3. Current Consumption

**Figure 6.**
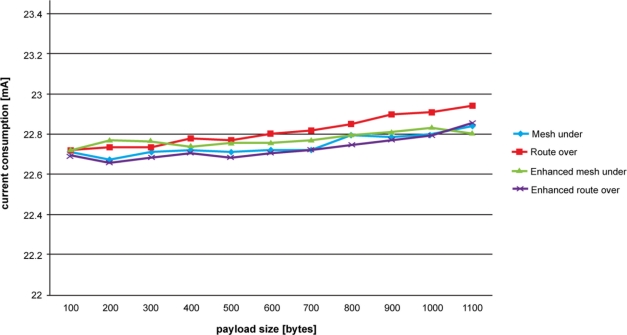
Current consumption evolution according to ICMP payload size. Hop-by-hop fragment reassembling performed by route over proves to be energy demanding. The control on packet forwarding introduced in controlled mesh under, slightly increases current consumption compared with mesh under.

Figure 6 shows the results obtained for current consumption. The tests were performed by measuring the current drawn by a node forwarding ping requests and replies in a two-hop network. We were interested in measuring the current drawn by the relay node in receiving, processing and sending fragments. For each different payload size, we ran tests sampling the current consumption each 0.02 ms. The values obtained refer to the average current consumed by the relay node from the time a fragment is received up to its transmission to the next-hop. The reported average values have a confidence level of 95%. Current drawn in inactivity states was not taken into account. The device used for these measures is the Agilent Technologies DC power Analyzer N67705A.

As expected, a node adopting a route over technique consumes more energy than others. Once again, hop-by-hop fragment reassembling proves to be costly in constrained network environment. This can be appreciated by comparing the route over performance with that of its enhanced scheme. In fact, enhanced route over simplifying the fragment forwarding significantly reduces the current drawn by the node. Enhanced route over gives the best performance in terms of current consumption. It should be pointed out that since enhanced route over is the default routing technique of Blip; it is optimized in order to work with it. Consequently, enhanced route over is inclined to work better on Blip.

Mesh under has a performance very similar to that of enhanced route over. Although affected by a large number of retransmissions, by avoiding complex fragment processing mesh under maintains low current consumption. However, in comparison with the less complex mesh under, the augmented process complexity of controlled mesh under does not significantly increase current consumption. Here, the augmented complexity is aimed at the control associated to fragment forwarding. Nevertheless, considering the overall current consumption and taking as an example a network composed of many nodes, better management of the controlled mesh bandwidth would result in energy-saving. In fact, in controlled mesh under, forwarding of unnecessary fragments would be avoided and the nodes would be subjected to a lower workload.

Finally, we observed that standard deviation is very low for all the forwarding schemes, and consequently deemed not relevant for the energy consumption results.

## 5. Conclusions

In this paper, we analyze the performances of the routing schemes used in 6LoWPAN networks and propose a new scheme. 6LoWPAN defines two routing strategies: mesh under and route over. Furthermore, with these techniques we propose an alternative mesh under scheme in order to improve the critical aspects of the mesh under forwarding. We call our proposal controlled mesh under. In our evaluation we also include an alternative route over scheme that we call enhanced route over. This routing scheme is the default solution used in Blip. We implement and test route over, mesh under and controlled mesh under. The analyzed performance is carried out in a real 6LoWPAN network. Our purpose is to study the forwarding strategies of the different routing solutions when dealing with an IP fragmented packet. In a previous work we analyzed mesh under and route over in absence of IP packet fragmentation. Results can be found in [7].

The application domain where 6LoWPAN is to be deployed plays a substantial role in choosing which forwarding solution to adopt. The high packet loss experienced in mesh under does not make it recommendable for use in applications requiring a high degree of reliability. Route over is suggested for these critical application. First, controlled mesh under or, secondly, enhanced route over can both ensure reliability, but for a smaller payload range than that of route over. Controlled mesh under lowers the packet loss with respect to mesh under by providing a better bandwidth management. Actually, packet loss percentage is quite high for all the considered routing solution when the payload size is large. For these large payloads, we observe a rapid worsening of the 6LoWPAN performances, regardless of the forwarding technique used. In particular, route over experiences memory congestion problems for payload greater than 800 bytes. A better memory management could solve this problem and augment the route over reliability for high payload size.

Applications requiring a good latency performance should implement a controlled mesh under or enhanced route over solution. Mesh under has an acceptable latency performance, but lower as compared with controlled mesh under in the case of end-to-end delay time. However, applications generating low traffic and small packet size could also implement a mesh under scheme.

Energy consumption is a crucial factor in sensor networks. Sensors are usually power supply constrained in, since they are battery powered. Our studies demonstrate that routing solutions subjected to a higher workload have a poor behavior in terms of consumed energy. In this sense, although subjected to a lower packet loss and to less fragment retransmission, route over consumes more current than the other routing solutions. Its alternative solution, that is, enhanced route over, lowers current consumption by avoiding hop-by-hop fragment reassembling.

Fragment retransmission is another crucial aspect in energy consumption. It is well known that the peak in energy consumption is located in the transmission state. As a consequence, those routing solutions characterized by a high retransmission rate spend more time in the transmission state and are inclined to waste more energy than other techniques. In this paper, mesh under turned out to be the technique subjected to the highest number of retransmissions. Mesh under compensates energetically for the cost of retransmissions with its energetically efficient fragment processing. As result, mesh under shows good performance in terms of energy consumption. As regards controlled mesh under, the control added to monitor fragment forwarding requires a slight increase of energy compared with mesh under. Furthermore, the better usage of the communication channel allowed by controlled mesh under lowers the overall network current consumption by avoiding the propagation of useless fragments.

In conclusion, route over proves to be more robust to packet loss, but less energy saving than the other routing schemes. Weaknesses of route over are also found in the high latency experienced in packet transmission. Enhanced route over proves to be capable of solving these limitations in latency and energy performance, but is unable to maintain the packet loss to the same degree as route over. Both mesh techniques show a good performance in terms of latency and energy consumption, with controlled mesh under yielding a better result in the end-to-end delay performance. While increasing the complexity of fragment forwarding, controlled mesh under does not result in a significant growth of the consumed current. The high packet loss shown in mesh under decreases in controlled mesh under thanks to better management of the channel bandwidth.

Future works will include further research on the 6LoWPAN routing scheme. In particular, we plan to study fragments retransmission with the aim of finding a possible solution to reduce their number. We expect to improve the general performances of the 6LoWPAN network and, in particular, to reduce energy consumption.

## Figures and Tables

**Table 1.** Packet loss percentage. Route over proves to be more robust to packet loss than the other techniques. However, starting from a payload size of 900 bytes, buffer congestion causes a rapid worsening of route over packet loss. Link retransmissions due to collisions are the main cause of packet loss for mesh under, controlled mesh under and enhanced route over.

**Payload size [bytes]**	**Route over**	**Mesh under**	**Controlled mesh under**	**Enhanced route over**
100	0%	0%	0%	0%
200	0%	0%	0%	1%
300	0%	4%	2%	5%
400	3%	15%	4%	6%
500	3%	21%	10%	13%
600	2%	27%	20%	16%
700	3%	32%	24%	23%
800	3%	37%	28%	29%
900	33%	35%	33%	34%
1,000	49%	42%	35%	31%
1,100	58%	48%	41%	41%
